# Just-in-Time Adaptive Interventions to Reduce Sedentary Behavior and Promote Physical Activity in Patients With Chronic Disease: Scoping Review

**DOI:** 10.2196/81378

**Published:** 2026-08-07

**Authors:** Junchen Guo, Furong Chen, Chan Chen, Yunyun Dai, Xianghua Xu, Yongyi Chen, Mengying Huang

**Affiliations:** 1 Australasian Health Outcomes Consortium, University of Wollongong, Wollongong, NSW, Australia; 2 Department of Palliative Care, Hunan Cancer Hospital, Changsha, Hunan, China; 3 School of Nursing, Guangzhou Medical University, Guangzhou, China; 4 School of Journalism and Communication, Hunan Normal University, Changsha, China; 5 School of Nursing, Guilin Medical University, Guilin, China; 6 Department of Nursing, Hunan Provincial Hospital of Integrated Traditional Chinese and Western Medicine, No. 58 Lushan Road, Yuelu District, Changsha, Hunan, 410006, China, 86 17373566909

**Keywords:** physical activity, sedentary behavior, just-in-time adaptive intervention, digital health, chronic disease

## Abstract

**Background:**

Patients with chronic diseases often struggle to maintain sufficient physical activity (PA) and reduce sedentary behavior in daily life. Just-in-time adaptive interventions (JITAIs), delivered through mobile health technologies such as mobile apps, wearable devices, sensors, and ecological momentary assessment, offer timely and personalized support based on individuals’ changing needs. However, the evidence base for their use in promoting PA and reducing sedentary behavior among patients with chronic diseases remains fragmented.

**Objective:**

This review aimed to describe study characteristics and design features of JITAIs, summarize reported evaluation findings related to PA and sedentary behavior, and identify barriers and facilitators related to engagement and use of JITAIs.

**Methods:**

A comprehensive literature search was conducted in PubMed, Embase, Web of Science, CINAHL, and Scopus from database inception to June 2025. Two researchers independently screened the records, selected eligible studies, and performed data charting to ensure rigor and consistency.

**Results:**

A total of 14 studies were included in this review. Of these, 6 primarily targeted PA, 1 focused solely on sedentary behavior, and 5 addressed both PA and sedentary behavior. Of the remaining, 6 studies provided preliminary evidence supporting the positive effects of JITAIs in promoting PA among individuals with chronic diseases, while 3 studies suggested positive effects on reducing sedentary behavior. Barriers and facilitators influencing the use of JITAIs in this population were identified. Barriers included technological usability and literacy, burden and intrusiveness of the intervention, perceived value and acceptability, external and contextual barriers, and privacy and data security concerns. Facilitators included enhanced motivation and behavioral engagement, increased awareness and self-monitoring, usability and integration into daily life, timely and personalized support, and support from health care professionals and the care environment.

**Conclusions:**

This scoping review identified preliminary evidence suggesting that JITAIs may help reduce sedentary behavior and promote PA among patients with chronic diseases. However, more large-scale, high-quality randomized controlled trial studies are needed to strengthen evidence and generalizability.

## Introduction

According to the World Health Statistics 2023 report published by the World Health Organization, noncommunicable diseases account for approximately 41 million deaths annually, representing 74% of all deaths worldwide [1]. Chronic diseases, such as cardiovascular diseases, chronic respiratory diseases, and diabetes, are long-term conditions that generally require ongoing medical care and sustained self-management [2]. These conditions can often be prevented or managed through behavioral changes, particularly by increasing physical activity (PA) and reducing sedentary behavior. PA refers to any bodily movement produced by skeletal muscles that results in energy expenditure, whereas sedentary behavior refers to any waking behavior characterized by low energy expenditure while sitting, reclining, or lying [3,4]. Regular PA is associated with improved cardiovascular health, glycemic control, and mental well-being [5,6]. However, many patients with chronic diseases have difficulty achieving sufficient levels of PA and limiting sedentary time in daily life. Prolonged sedentary behavior has been associated with impaired metabolic function, obesity, anxiety, depression, and increased risks of hypertension, diabetes, cardiovascular disease, and other adverse health outcomes [7-9]. Therefore, reducing sedentary behavior and promoting PA are important components of chronic disease management.

Traditional lifestyle interventions designed to reduce sedentary behavior and promote PA may be effective in controlled settings, but they often rely on standardized, fixed plans that deliver the same type and intensity of support to all patients at predetermined time points [10]. This approach may overlook individual variability and dynamic changes in patients’ needs, potentially resulting in insufficient, excessive, or poorly matched support [10]. Digital health, which refers to the use of digital technologies to support health care delivery, monitoring, and self-management, offers opportunities to provide more timely and personalized behavioral support [11]. Within digital health, mobile health (mHealth) interventions use mobile phones, wearable activity trackers, sensors, and other wireless technologies to monitor health behaviors and deliver intervention content in everyday settings [12]. In interventions targeting PA and sedentary behavior, mHealth tools can track steps, activity intensity, sedentary time, and location, as well as deliver feedback, reminders, motivational messages, and activity suggestions [13].

In recent years, just-in-time adaptive interventions (JITAIs) have emerged as a promising mHealth approach to supporting behavior change [14]. A JITAI is designed to provide the right type and amount of support at the right time by adapting intervention delivery to a person’s current needs, context, or state of vulnerability and opportunity [14,15]. In practice, JITAIs commonly use data from wearable sensors, mobile apps, ecological momentary assessment (EMA), or contextual information, such as time of day, location, and recent activity patterns, to determine when and how support should be delivered [15,16]. Key JITAIs design components typically include decision points, tailoring variables, intervention options, decision rules, and proximal or distal outcomes [16]. Previous studies have applied JITAIs across a range of health behavior areas, including smoking cessation, alcohol reduction, dietary improvement, and mental health support, suggesting that they may provide more personalized and timely support in daily life than traditional interventions [17,18]. In the field of PA promotion and sedentary behavior reduction, JITAIs have increasingly been applied among individuals with diabetes, cardiovascular disease, and other long-term conditions [19-21]. A key potential advantage of JITAIs is their ability to use data from mobile phones, sensors, and self-reports to deliver context-sensitive support that accounts for daily fluctuations in symptoms, motivation, routines, and opportunities for PA [19-21].

Despite these potential advantages, the evidence base for the use of JITAIs to promote PA and reduce sedentary behavior among patients with chronic diseases remains fragmented. Existing studies also showed their implementation may be affected by potential disadvantages, as JITAIs often depend on patients’ access to and ability to use mobile or wearable technologies, which can create barriers related to technical problems, device compatibility, and user burden [15,16]. Frequent or poorly timed prompts may also lead to notification fatigue or disengagement [15,16]. How these advantages and disadvantages influence PA and sedentary behavior outcomes among patients with chronic diseases has not been consistently synthesized across existing studies. Moreover, current studies also differ in their design and evaluation outcomes, making it difficult to evaluate the effectiveness of JITAIs in promoting PA and reducing sedentary behavior in this population. Therefore, we conducted this scoping review focusing on studies in which JITAIs designed to reduce sedentary behavior and/or promote PA among patients with chronic diseases. The review aimed to describe the study characteristics and design features of JITAIs, summarize reported evaluation findings related to PA and sedentary behavior, and identify barriers and facilitators related to engagement and use of JITAIs. By mapping this evidence, the review may inform the development of future JITAIs that are more acceptable and suitable for supporting PA promotion and sedentary behavior reduction in chronic disease self-management.

## Methods

### Study Design

This scoping review was systematically conducted following the methodological framework proposed by the Joanna Briggs Institute (JBI) [22]. The review was reported in accordance with the PRISMA-ScR (Preferred Reporting Items for Systematic Reviews and Meta-Analyses Extension for Scoping Reviews) checklist (Checklist 1) [23]. Ethical approval was not required for this scoping review, as no new data were collected.

### Research Questions

The research questions of this scoping review were identified as follows:

- What are the key study characteristics and design features of JITAIs designed to reduce sedentary behavior and promote PA in patients with chronic disease?
- What evidence exists regarding the evaluation of these JITAIs in terms of PA and sedentary behavior?
- What barriers and facilitators have been reported in relation to the engagement and use of JITAIs among patients with chronic disease?

### Search Methods

#### Eligibility Criteria

The eligibility criteria were established based on the population-concept-context (PCC) framework proposed by the JBI [22]. The inclusion criteria were as follows: (1) population: studies involving adults (aged 18 years and older) diagnosed with one or more chronic diseases; (2) concept: studies that used JITAIs aimed at reducing sedentary behavior and/or promoting PA, where JITAIs were defined as interventions that use sensor technologies (eg, accelerometers, GPS, and light sensors) embedded in mobile devices to collect real-time behavioral and contextual data and deliver tailored support at moments when behavior change is most likely to occur [17]; (3) context: studies conducted in a variety of settings, including health care, community, and home settings, where JITAIs were used to support behavior change in individuals with chronic conditions; and (4) types of evidence sources: both quantitative and qualitative studies were included. Mixed methods studies were also eligible. Only peer-reviewed articles published in English were considered. Studies were excluded if they focused solely on healthy populations, used nonadaptive or nondigital interventions, or did not provide sufficient detail on the intervention characteristics or outcomes of interest.

#### Search Strategy

In collaboration with two independent university medical librarians in China, a systematic search was conducted across 5 electronic databases, including PubMed, Embase, Web of Science, CINAHL, and Scopus, covering literature from database inception to June 2025. An initial exploratory search was performed in PubMed to identify relevant keywords and MeSH terms based on titles and abstracts of pertinent studies. Boolean operators were used to capture studies related to the 4 core concepts: “just-in-time adaptive interventions,” “physical activity,” “sedentary behavior,” and “chronic disease.” To define the scope of chronic conditions, 10 diseases were selected based on the list published by the Office of the Assistant Secretary for Health of the United States [24]: hypertension, type 2 diabetes (T2D), chronic obstructive pulmonary disease, stroke, heart failure, coronary artery disease, chronic kidney disease, hyperlipidemia, asthma, and arthritis. Conditions that were psychiatric (eg, schizophrenia), acute, or not conducive to self-management (eg, dementia) were excluded as this review focused on chronic physical conditions in which PA and sedentary behavior can be targeted through patient-led or patient-engaged self-management [25]. These conditions may require different intervention approaches or rely more heavily on management led by clinicians or caregivers, and were therefore beyond the scope of this review. Additionally, a snowballing technique was used to manually screen the reference lists of included studies to identify further relevant literature. The PubMed search strategy is presented in Table 1, while the complete search terms, strategies, and results for other databases are provided in Multimedia Appendix 1.

**Table 1.** Search strategy of PubMed.

Procedure	Search strategy
#1	“chronic disease” [MeSH Terms] OR “essential hypertension” [MeSH Terms] OR “heart failure” [MeSH Terms] OR “coronary artery disease” [MeSH Terms] OR “stroke” [MeSH Terms] OR “paresis” [MeSH Terms] OR “arthritis” [MeSH Terms] OR “osteoarthritis” [MeSH Terms] OR “asthma” [MeSH Terms] OR “renal insufficiency, chronic” [MeSH Terms] OR “pulmonary disease, chronic obstructive” [MeSH Terms] OR “diabetes mellitus” [MeSH Terms] OR “insulin resistance” [MeSH Terms] OR “hyperlipidemias” [MeSH Terms]
#2	“chronic disease” [Title/Abstract] OR “chronic illness” [Title/Abstract] OR “chronic condition” [Title/Abstract] OR “non communicable disease” [Title/Abstract] OR “noncommunicable disease” [Title/Abstract] OR “chronically ill” [Title/Abstract] OR “hypertension” [Title/Abstract] OR “hypertensive” OR “high blood pressure” [Title/Abstract] OR “heart failure” [Title/Abstract] OR “CHF” [Title/Abstract] OR “HF” [Title/Abstract] OR “cardiac failure” [Title/Abstract] OR “heart decompensation” [Title/Abstract] OR “coronary artery disease” [Title/Abstract] OR “coronary arteriosclerosis” [Title/Abstract] OR “coronary atherosclerosis” [Title/Abstract] OR “angina pectoris” [Title/Abstract] OR “CAD” [Title/Abstract] OR “heart disease” [Title/Abstract] OR “myocardial infarction” [Title/Abstract] OR “unstable angina*” [Title/Abstract] OR “angor pectoris” [Title/Abstract] OR “coronary thrombosis” [Title/Abstract] OR “acute coronary syndrome” [Title/Abstract] OR “myocardial ischemia” [Title/Abstract] OR “myocardial ischaemia” [Title/Abstract] OR “stroke” [Title/Abstract] OR “hemiplegia” [Title/Abstract] OR “hemiplegia” [Title/Abstract] OR “hemiplegias” [Title/Abstract] OR “paresis” [Title/Abstract] OR “cerebrovascular trauma” [Title/Abstract] OR “cerebrovascular accident” [Title/Abstract] OR “CVA” [Title/Abstract] OR “apoplexy” [Title/Abstract] OR “arthritis” [Title/Abstract] OR “rheuma*” [Title/Abstract] OR “osteoarthritis” [Title/Abstract] OR “arthritides” [Title/Abstract] OR “polyarthritis” [Title/Abstract] OR “polyarthritides” [Title/Abstract] OR “asthma” [Title/Abstract] OR “status asthmaticus” [Title/Abstract] OR “bronchial hyper reactivity” [Title/Abstract] OR “asthmatic” [Title/Abstract] OR “wheez” [Title/Abstract] OR “bronchial” [Title/Abstract] OR “obstructive lung disease” [Title/Abstract] OR “chronic renal insufficiency” [Title/Abstract] OR “chronic kidney failure” [Title/Abstract] OR “chronic renal failure” [Title/Abstract] OR “chronic renal disease” [Title/Abstract] OR “chronic kidney disease” [Title/Abstract] OR “chronic kidney disorder” [Title/Abstract] OR “CKD” [Title/Abstract] OR “ESRD” [Title/Abstract] OR “CRD” [Title/Abstract] OR “chronic kidney insufficiency” [Title/Abstract] OR “chronic obstructive pulmonary disease” [Title/Abstract] OR “chronic bronchitis” [Title/Abstract] OR “COPD” [Title/Abstract] OR “chronic obstructive airway disease” [Title/Abstract] OR “chronic airflow obstruction” [Title/Abstract] OR “chronic obstructive lung disease” [Title/Abstract] OR “emphysema” [Title/Abstract] OR “diabetes mellitus” [Title/Abstract] OR “insulin resistance” [Title/Abstract] OR “DMII” [Title/Abstract] OR “DM2” [Title/Abstract] OR “noninsulin dependent” [Title/Abstract] OR “impaired glucose tolerance” [Title/Abstract] OR “impaired glucose tolerant” [Title/Abstract] OR “hyperlipidemias” [Title/Abstract] OR “overweight” [Title/Abstract] OR “obese” [Title/Abstract]
#3	#1 OR #2
#4	“motor activity” [MeSH Terms] OR “exercise” [MeSH Terms] OR “walking” [MeSH Terms] OR “sports” [MeSH Terms] OR “jogging” [MeSH Terms] OR “swimming” [MeSH Terms] OR “health behaviour” [MeSH Terms] OR “cool-down exercise” [MeSH Terms] OR “gymnastics” [MeSH Terms] OR “muscle stretching exercises” [MeSH Terms] OR “circuit-based exercise” [MeSH Terms] OR “endurance training” [MeSH Terms] OR “high-intensity interval training” [MeSH Terms] OR “plyometric exercise” [MeSH Terms] OR “resistance training” [MeSH Terms] OR “warm-up exercise” [MeSH Terms] OR “weight lifting” [MeSH Terms] OR “screen time” [MeSH Terms]
#5	“physical activit*” [Title/Abstract] OR “physical fitness” [Title/Abstract] OR “sedentary behaviour” [Title/Abstract] OR “sitting” [Title/Abstract] OR “sedentariness” [Title/Abstract] OR “sedentar*” [Title/Abstract] OR “walk*” [Title/Abstract] OR “run” [Title/Abstract] OR “sport*” [Title/Abstract] OR “jog” [Title/Abstract] OR “swim*” [Title/Abstract] OR “cool-down exercise” [Title/Abstract] OR “cool down exercise” [Title/Abstract] OR “gymnastics” [Title/Abstract] OR “muscle stretching exercises” [Title/Abstract] OR “circuit-based exercise” [Title/Abstract] OR “endurance training” [Title/Abstract] OR “high-intensity interval training” [Title/Abstract] OR “high intensity interval train*” [Title/Abstract] OR “plyometric exercise” [Title/Abstract] OR “resistance training” [Title/Abstract] OR “resistance train*” [Title/Abstract] OR “warm-up exercise” [Title/Abstract] OR “weight lift*” [Title/Abstract] OR “circuit train*” [Title/Abstract] OR “circuit exercise*” [Title/Abstract] OR “weight train*” [Title/Abstract] OR “aerobic train*” [Title/Abstract] OR “aerobic exercise*” [Title/Abstract] OR “cardio train*” [Title/Abstract] OR “screen behavio*” [Title/Abstract] OR “screen Time” [Title/Abstract] OR “screen watching” [Title/Abstract] OR “TV watching” [Title/Abstract] OR “prolonged sitting” [Title/Abstract] OR “lying time” [Title/Abstract] OR “television viewing” [Title/Abstract] OR “active recreation” [Title/Abstract] OR “active leisure” [Title/Abstract] OR “active living” [Title/Abstract]
#6	#4 OR #5
#7	“Just-in-time adaptive intervention” [Title/Abstract] OR “Just-in-time” [Title/Abstract] OR “JITAI” [Title/Abstract] OR “ecological momentary intervention*” [Title/Abstract] OR “EMI” [Title/Abstract] OR “adaptive intervention” [Title/Abstract] OR “dynamic intervention” [Title/Abstract] OR “real time intervention*” [Title/Abstract] OR “context aware*” [Title/Abstract] OR “context triggered” [Title/Abstract] OR “context tailor*” [Title/Abstract] OR “dynamic tailor*” [Title/Abstract] OR “real time tailor*” [Title/Abstract] OR “sensor triggered” [Title/Abstract] OR “geofenc*” [Title/Abstract] OR “context sens*” [Title/Abstract] OR “real time context*” [Title/Abstract] OR “persuasive technolog*” [Title/Abstract] OR “sensing technolog*” [Title/Abstract]
#8	#3 AND #6 AND #7

#### Data Management

##### Data Selection

The search results were imported into EndNote software (version X9.1; Clarivate). After removing duplicate records, two researchers (JG and FC) independently screened the titles and abstracts of all retrieved studies to assess initial eligibility based on the predefined inclusion criteria. Studies that met the inclusion criteria during title and abstract screening were retrieved in full text and further assessed for eligibility. Any discrepancies between the two primary researchers were resolved through discussion or adjudicated by the third reviewer (YC).

##### Data Extraction

Two researchers (JG and MH) independently extracted and recorded data using a predesigned standardized Excel form (Microsoft, 2022). The data extraction process was guided by the review’s research questions and included the following information: (1) characteristics of included studies such as first author, year of publication, country, objectives, study design, population and setting, and sample size; (2) design characteristics of JITAIs, including theoretical framework, target behaviors, sensors/data sources, tailoring variables, decision points, intervention options, decision rules, prompt frequency/timing, adaptation mechanisms, intervention duration, engagement, and implementation-related measures; (3) evaluation of JITAIs in terms of PA and sedentary behavior; and (4) reported barriers and facilitators related to engagement and use of JITAIs. A third reviewer (YD) independently reviewed the extracted data from all included studies. Any discrepancies between the two primary researchers were resolved through discussion or adjudicated by the third reviewer (YD).

##### Data Synthesis and Analysis

Data synthesis and analysis were conducted using a descriptive and narrative approach, in line with the objectives and research questions of this scoping review. Given the heterogeneity of study designs and target populations, a meta-analysis was not conducted. Data on study characteristics, design features of JITAIs, and evaluation findings were descriptively synthesized and tabulated to summarize the overall profile of the included studies [26]. Where appropriate, similar JITAI design components were grouped into categories. For example, decision rules were categorized as EMA-based if-then rules, sensor-triggered or sedentary-threshold rules, scheduled rules, microrandomization rules, or coach- or staff-supported rules. Additionally, qualitative data and open-ended data were synthesized using inductive thematic analysis to identify reported barriers and facilitators related to the engagement and use of JITAIs among patients with chronic diseases [27]. Two reviewers (JG and FC) independently coded relevant qualitative findings and extracted statements. Codes were compared, refined, and grouped into overarching themes and subthemes. Discrepancies in interpretation were resolved through discussion or adjudicated by the third reviewer (YD). Final themes and findings were reviewed by the full research team to ensure coherence, transparency, and consistency with the review questions.

## Results

### Search Results

**Figure 1.**
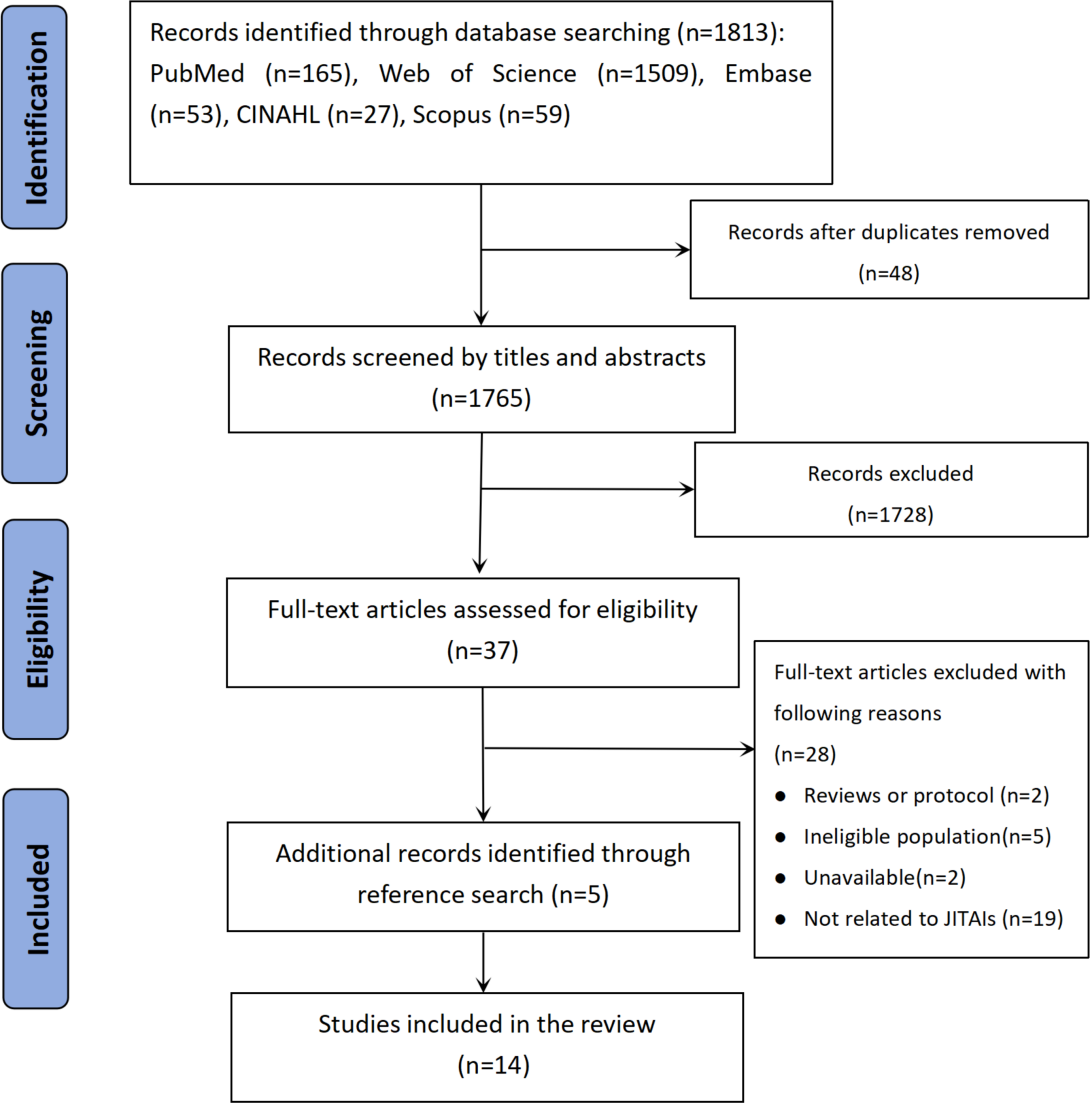
PRISMA (Preferred Reporting Items for Systematic Reviews and Meta-Analyses) flow diagram of study selection. JITAI: just-in-time adaptive intervention.

A total of 1813 records were identified through searches of 5 electronic databases. After removing 48 duplicates, 1765 records remained for title and abstract screening, of which 1728 were excluded based on relevance. The full texts of the remaining 37 articles were assessed for eligibility, resulting in the exclusion of 28 studies. An additional 5 relevant studies were identified through reference list screening. Finally, 14 studies were included in this scoping review. The PRISMA flow diagram of study selection is shown in Figure 1.

### Characteristics of Included Studies

The publication years of included studies ranged from 2015 to 2025. The majority (n=9) were conducted in the United States [28-36], while the remaining 5 were carried out in Ireland [37], Australia [38], the Netherlands [19], Singapore [39], and the Czech Republic [20], respectively. All studies were described as feasibility or pilot studies, including 4 qualitative studies [19,31,37,38], 3 randomized controlled trial (RCT) studies [28,29,32], 6 single-armed feasibility or pilot studies [20,33-36,39], and 1 prospective cohort study [30]. Sample sizes ranged from 8 to 108 participants. Among those, 4 studies recruited individuals with T2D [19,20,35,38], 3 targeted individuals undergoing or completing cardiac rehabilitation [28,31,33], and 2 included individuals with stroke [30,37]. The remaining studies enrolled participants with hypertension (n=1) [29], spinal cord injury (n=1) [34], acute coronary syndrome or those who had undergone coronary revascularization (n=1) [33], individuals who were overweight or obese (n=1) [36], and older adults with a chronic condition (n=1) [39]. More details of the characteristics of included studies are presented in Table 2.

**Table 2.** Characteristics of included studies.

Author	Year of publication	Country	Objectives	Study design	Population and setting	Sample size
Hietbrink et al [19]	2025	Netherlands	To assess the acceptability of EMA^a^-driven, just-in-time adaptive lifestyle support in individuals with T2D^b^	Qualitative study	Population: people diagnosed with T2D Age: mean 70.5 (SD 9) years Setting: hospital	8
Cardy et al [37]	2024	Ireland	To explore the perspectives of people with strokes, their caregivers, and health care providers on the delivery of an adaptive, personalized mHealth^c^ intervention to promote PA^d^ after stroke	Qualitative study	Population: people with stroke, caregivers, health care workers Age: 54.5, 66.5, and 34 years Setting: hospital and community	People with stroke (n=12); caregivers (n=6); health care workers (n=10)
Golbus et al [28]	2024	USA	To support cardiac rehabilitation patients in increasing and sustaining PA using a mobile health intervention	RCT^e^	Population: people were enrolled in cardiac rehabilitation based on a qualifying diagnosis Age: mean 59.5 (SD 10.7) years Setting: medical and community health care center	108
Novak et al [20]	2024	Czech Republic	To describe the development and piloting of the mHealth intervention and its final version to be evaluated in the ENERGISED trial	Pilot study	Population: people diagnosed with prediabetes and T2D Age: between 40 and 76 years Setting: hospital	10
Bai et al [29]	2022	USA	To determine the effectiveness of wearable activity trackers alone or in combination with behavior change strategies for promoting PA among individuals with prehypertension or hypertension	RCT	Population: prehypertension or hypertension patients Age: mean 55 (SD 8.8) years Setting: medical clinics, community	44 (control: 22; intervention: 22)
Daryabeygi-Khotbehsara et al [38]	2022	Australia	To develop an Android mobile app to deliver a JITAI^f^ aimed at reducing sitting time and increasing PA in people with T2D	Qualitative study	Population: people diagnosed with T2D Age: mean 49.0 (SD 8.6) years Setting: not reported	10
Lau et al [30]	2022	USA	To evaluate the feasibility and acceptability of using accelerometry and EMA to monitor daily activity and symptoms in stroke survivors	Prospective cohort study	Population: people diagnosed with mild to moderate stroke Age: mean 52.8 (SD 7.48) years Setting: community	40
Mair et al [39]	2022	Singapore	To describe the development of JitaBug designed to support older adults to increase or maintain their PA level	Feasibility study	Population: community-dwelling older adults with chronic disease Age: mean 65.5 (SD 5.4) years Setting: community	31
Elnaggar et al [31]	2021	USA	To evaluate the acceptability of a wearable device, mobile app, and push messages to facilitate PA following cardiac rehabilitation completion	Qualitative study	Population: people with a previous cardiac event who qualified and were actively participating in cardiac rehabilitation Age: mean 66.7 (SD 8.6) years Setting: community	26
Park et al [32]	2021	USA	To examine preliminary effects of an mHealth intervention on group differences in PA and functional capacity to maintain exercise after cardiac rehabilitation	RCT	Population: people diagnosed with CVD^g^ and were within 2 weeks of completing cardiac rehabilitation Age: mean 66.7 (SD 8.6) years Setting: community	51 (control: 25; intervention: 26)
Sengupta et al [33]	2020	USA	To examine the usability and health behaviors of a prototypic mHealth intervention designed specifically for women with CHD^h^	Feasibility study	Population: women diagnosed with an acute coronary syndrome or coronary revascularization Age: mean 64.4 (SD 6.3) years Setting: medical center	10
Hiremath et al [34]	2019	USA	To use an mHealth-based PA measurement system to track PA levels of individuals with spinal cord injury in the community	Pilot study	Population: people diagnosed with spinal cord injury who were at least 6 months postinjury Age: mean 39.4 (SD 12.8) years Setting: community	20
Pellegrini et al [35]	2015	USA	To examine the acceptability of a new technology developed to interrupt prolonged bouts of sedentary time among adults with T2D	Feasibility study	Population: people diagnosed with T2D Age: mean 53.1 (SD 10.7) years Setting: community	8
Thomas et al [36]	2015	USA	To examine prompts and behavioral responses to the B-MOBILE JITAI for reducing sedentary behavior in overweight/obese individuals	Feasibility study	Population: overweight/obese participants with BMI≥25 kg/m^2^ Age: mean 47.5 (SD 13.5) years Setting: not reported	30

a EMA: ecological momentary assessment.

b T2D: type 2 diabetes.

c mHealth: mobile health.

d PA: physical activity.

e RCT: randomized controlled trial.

f JITAI: just-in-time adaptive intervention.

g CVD: cardiovascular disease.

h CHD: coronary heart disease.

### Design Characteristics of JITAIs

Among the included studies, 13 reported on the design characteristics of JITAIs, with 2 studies using the same version of the JITAIs [31,32], resulting in a total of 12 unique JITAIs. Additionally, 8 of the JITAIs were grounded in theoretical frameworks [19,20,29,32-34,38,39], with social cognitive theory being the most commonly applied [32,34]. Overall, 6 JITAIs [28,30,32-34,37] were designed to primarily target PA behavior, 1 focused solely on sedentary behavior [36], and 5 addressed both sedentary behavior and PA [20,29,35,38,39]. Most studies used wearable devices, smartphone apps, or both to collect PA or sedentary behavior data. Wearable or sensor-based data were used in studies (n=11) [20,28,29,31-36,38,38,39] using Fitbit or smartwatch devices, activity trackers, smartphone accelerometers, or external accelerometers. Several studies (n=4) also incorporated EMA or self-report data. Additional contextual data, such as location, weather, time of day, or day of week, were used in Hietbrink et al [19], Golbus et al [28], Daryabeygi-Khotbehsara et al [38], Mair et al [39], and Novak et al [20]. Two studies [31,32] also included app-entered clinical or health data, such as symptoms, blood pressure, heart rate, medication use, or self-reported health measures.

Tailoring variables could be grouped into 5 categories: behavioral variables, contextual variables, self-reported momentary states, goal/progress variables, and clinical or safety-related variables. Behavioral variables included step count, sedentary bout duration, walking status, activity intensity, and recent PA patterns [20,28,29,31-36,38,39]. Contextual variables, including time of day, weather, location, weekday/weekend, or study phase [19,20,28,38,39]. Self-reported momentary states, such as mood, fatigue, restrictions, cravings, readiness, and energy [19,33,34]. Goal- or progress-related tailoring was used in 6 studies [20,29,33,34,38,39]; for example, Novak et al [20] tailored messages according to recent step counts, weekly step goals, and individual action plans. Clinical or safety-related tailoring, such as symptom-triggered safety responses, was mainly reported in Elnaggar et al [31] and Park et al [32].

Decision points also differed across studies. Some interventions (n=4) [20,34-36] used sensor-triggered decision points, where prompts were delivered when a behavioral threshold was detected. For example, Pellegrini et al [35] delivered prompts after 20 minutes of consecutive sedentary time; Thomas et al [36] tested prompts after 30, 60, or 120 minutes of sedentary behavior; Novak et al [20] used Fitbit-detected inactivity or walking patterns; and Hiremath et al [34] triggered feedback when moderate-intensity wheelchair-based activity was detected. Other studies (n=5) [28,31,32,38,39] used scheduled or semi-scheduled decision points. Golbus et al [28] and Daryabeygi-Khotbehsara et al [38] also used microrandomized decision points, where participants could be randomized to receive or not receive an intervention message at predefined times or contexts. EMA-based decision or assessment points were used in Hietbrink et al [19] and Sengupta et al [33].

Intervention options could be grouped into PA prompts (n=9) [19,20,28,31-34,38,38,39] sedentary break prompts (n=5) [20,35,36,38,39], goal-setting or planning support (n=7) [20,28,29,33,34,38,39], feedback and encouragement (n=8) [19,20,29,31-34,39], education or coping support (n=5) [19,20,31-33], and safety prompts, which were specifically reported in Elnaggar et al [31] and Park et al [32], where participants reporting symptoms such as chest pain or shortness of breath received safety-related guidance. Decision rules were mostly simple and rule-based, which could be classified as EMA-based if-then rules, sensor-triggered if-then rules, sedentary-threshold rules, scheduled rules, microrandomization rules, and coach- or staff-supported rules. EMA-based if-then rules were used in 2 studies. Hietbrink et al [19] matched text messages to EMA-reported activity, mood, location, restrictions, weather, or cravings, while Sengupta et al [33] used EMA and goal information to trigger reminders or reinforcement messages. Four studies [20,34-36] adopted sensor-triggered or sedentary-threshold rules, where prompts were delivered after predefined behavioral states such as inactivity, low-intensity walking, moderate-intensity wheelchair activity, or prolonged sedentary time. Scheduled rules were used in 4 studies [20,31,32,39], with messages delivered at fixed daily or weekly times. Probability-based or availability-based microrandomization was used in Golbus et al [28] and Daryabeygi-Khotbehsara et al [38], where participants were randomized to receive or not receive messages at predefined decision points, sometimes conditional on availability. Four studies [29,31-33] used coach- or staff-supported decision rules, where participant data were reviewed by coaches or study staff to support tailored feedback, motivational messages, or safety guidance.

The adaptation mechanisms could be grouped into sensor-based tailoring, contextual tailoring, self-report-based tailoring, goal/progress-based tailoring, and coach- or staff-supported tailoring in this study. Sensor-based tailoring was used in 6 studies [20,34-36,38,39], which adapted messages according to step count, sedentary time, activity intensity, or walking patterns. Contextual tailoring based on time, weather, location, or day of week was used in 5 studies [19,20,28,38,39]. Two studies [19,33] adopted self-report-based tailoring, which used EMA responses such as mood, fatigue, restrictions, cravings, or readiness for activity. Goal/progress-based tailoring (n=6) [20,29,33,34,38,39] adapted feedback according to whether participants were meeting activity or sitting goals. Four studies [29,31-33] used coach- or staff-supported tailoring, which involved study staff or health coaches reviewing participant data and providing individualized feedback. Furthermore, a total of 12 studies reported intervention duration of JITAIs, which ranged from 7 days to 6 months, and was typically 2 to 12 weeks. Further details regarding the design characteristics of the JITAIs are provided in Table 3.

**Table 3.** Design characteristics of JITAIs^a^.

Study	Theoretical framework	Target behavior	Sensors/data sources	Tailoring variables	Decision points	Intervention options	Decision rules	Prompt frequency/timing	Adaptation mechanisms	Intervention duration
Hietbrink et al [19], 2025	Behavior maintenance	PA^b^	EMA^c^ self-report via SMS link/mobile phone. No passive wearable sensor was used. Participants completed EMAs on activity, location, mood, condition/restrictions, weather, and cravings. Baseline web-based survey measured self-efficacy and phase of behavior change.	Activity, location, mood, physical/mental restrictions, weather, cravings; plus baseline self-efficacy and phase of behavior change used to select suitable messages.	Each EMA prompt functioned as a decision point. EMAs were delivered at 1‐2 semirandom time points per day: once in the morning and once in the afternoon/evening.	Lifestyle support messages, including PA prompts, motivational/reflective messages, skill-building tips, and coping support	EMA-based if-then rules	EMA prompts 1‐2 times/day; intervention options delivered by text message, maximum 2 intervention messages/day.	Contextual tailoring and self-report-based tailoring	2 weeks
Golbus et al [28], 2024	Not reported	PA	Smartwatch data from Apple Watch or Fitbit Versa; mobile study app; step count measured after message delivery. Contextual data included weather, time of day, day of week, and time since enrollment.	Weather, temperature/precipitation, time of day, weekday/weekend, duration in study. Some messages could include preferred name, gain/loss framing, emoji, or dashboard hyperlink.	Participants were randomized 4 times/day to receive either no message or a PA-promoting message.	PA-promoting text messages or no message, with additional exercise planning, feedback, and self-monitoring components	Probability-based microrandomization	4 activity-message decision points/day over the trial; expected activity message rate ≈1/day due to 25% probability at each point.	Contextual tailoring	6 months
Novak et al [20], 2024	Self-regulation theory	PA and sedentary behavior	Fitbit Inspire 2 activity tracker; HealthReact platform; Fitbit step count, walking cadence proxy, heart rate to confirm wear, and syncing pattern.	Fitbit-detected walking/sitting patterns, step count, heart rate/wear status, recent step counts, weekly step goal progress, individual action plans/routines, baseline step goals, Fitbit syncing reliability, phone type, internet access/mobile data availability.	Main decision points included 5 min walking with 60‐100 steps/min between 8 AM and 8 PM; 30 min with zero steps plus heart rate detection between 4 and 8 PM. Other decision points were individual routine-based reminders and fixed weekly times.	PA prompts, sedentary break prompts, action-plan reminders, goal review, feedback/encouragement, and health education	Sensor-triggered if-then rules plus scheduled rules	Expected 3‐6 messages/week. Caps: Stand Up max 1/day; Walk Faster max 2/day with ≥60 min interval. Goal review: Friday 8‐10 PM; Feedback/encouragement: Sunday 6‐8 PM; Health education: Tuesday 6‐8 PM.	Sensor-based tailoring, contextual tailoring, and goal/progress-based tailoring	2 weeks
Bai et al [29], 2022	Motivational interviewing and self-determination theory	PA and sedentary behavior	Fitbit Charge HR 3/Fitbit device for self-monitoring; ActiGraph wGT3X-BT used to assess MVPA^d^, steps, and sedentary time.	Fitbit-derived activity progress; step counts; PA goals; goal review information used by the health coach.	Not reported.	Self-monitoring, goal setting/review, adaptive feedback	Coach-led goal/feedback rules	Not reported	Goal/progress-based tailoring	12 weeks
Daryabeygi-Khotbehsara et al [38], 2022	Behavior change techniques	PA and sedentary behavior	iMOVE Android app; SORD wearable sensor for real-time sitting, standing, and walking detection; Bluetooth connection; smartphone GPS for location; OpenWeather API for weather; Android time data; cloud server/database.	Sitting/standing/walking status; location, especially home vs workplace; weather conditions; time of day; weekday/weekend; personal sitting and walking goals.	Five daily decision windows included morning, lunchtime, afternoon 1, afternoon 2, and evening, from 8 AM to 8:30 PM. Randomization occurred only when participants were available: not driving, not sleeping, not already active, and connected to the intern.	“Sit less/move more” prompts, goal setting	Availability-based microrandomization	5 decision points/day; 70% intervention probability, split into 35% Sit Less and 35% Move More; 30% no-message control.	Sensor-based tailoring, contextual tailoring, and goal/progress-based tailoring	Not reported
Lau et al [30], 2022	Not reported	PA	Accelerometer plus EMA surveys; retrospective self-report assessments; acceptability questionnaire.	Daily activity and health-related symptoms: mood, cognitive complaints, fatigue, and pain.	Not reported.	Monitoring only	Not reported	8 EMA surveys/day for 7 days.	Not reported	7 days
Mair et al [39], 2022	Behavior Change Wheel	PA and sedentary behavior	Fitbit Charge 4; JitaBug smartphone app; Firebase repository; Fitbit data repository; OpenWeather API; smartphone GPS/home postcode for weather; accelerometer/activity tracker data; EMA for mood and well-being; voice memos for contextual PA information.	Real-time PA level from Fitbit, chosen activity goal, step-count vs activity-minute goal, time of day, daily goal status, and weather condition.	Server checked Fitbit data every hour between 9 AM and 8 PM to determine goal achievement; personalized JITAI messages were generated at 12:30 PM, 5:30 PM, and 8:30 PM using Fitbit, Firebase, and weather data.	Goal feedback, PA suggestions, activity-break prompts, planning reminders	Rule-based if-then rules	Hourly goal-achievement checks from 9 AM to 8 PM; personalized messages at 12:30 PM, 5:30 PM, and 8:30 PM.	Sensor-based tailoring, contextual tailoring, and goal/progress-based tailoring	6 weeks
Elnaggar et al [31], 2021	Not reported	PA	Fitbit Charge 2; Fitbit app; Movn mobile app; Fitabase; self-entered daily weight, blood pressure, heart rate, medication use, uncaptured exercise, and CVD^e^ symptoms. Fitbit step count was the main PA data used.	Step count; PA progress; self-entered health data; cardiovascular symptom entries; staff knowledge of participant progress; participant-specific feedback needs.	Scheduled push-message times; Fitbit inactivity reminders after >2 hours of inactivity; symptom-triggered safety response when chest pain or shortness of breath was reported.	PA prompts, self-monitoring, education, symptom safety prompt	Scheduled + symptom/device-triggered rules	Push messages 3 times/week on random weekdays between 9 AM and 6 PM; Fitbit inactivity nudge after >2 hours of inactivity.	Coach- or staff-supported tailoring	2 months
Park et al [32], 2021	Social cognitive theory	PA	Fitbit Charge 2; Fitbit app; Movn mobile app; self-entered weight, blood pressure, heart rate, medication use, additional exercise, cardiovascular symptoms; and 6-minute walk test.	Step count, PA progress, self-entered health/activity data, cardiovascular symptoms, and study team–delivered motivational/educational content.	Scheduled push-message times; participant entries of health measures; symptom reporting through Movn; shortness of breath or chest pain triggered a safety prompt to call 911; study team triaged entries once daily.	Self-monitoring, PA prompts, education, symptom safety prompt	Scheduled + symptom/device-triggered rules	Push messages 3 times/week on random days between 9 AM and 6 PM; symptom safety prompt when chest pain or shortness of breath was reported.	Coach- or staff-supported tailoring	2 months
Sengupta et al [33], 2020	Behavior theory	PA	HerBeat smartphone app; Moto 360 smartwatch; smartwatch accelerometer/gyroscope-derived step count; heart rate; EMA surveys; cloud/server database; web-based health-coach dashboard. Data were refreshed on the dashboard every 10 min; smartwatch streamed step count and heart rate every 3 min from 6 AM to 10 PM.	Step count; distance walked; heart rate; walking goals; goal completion; readiness to begin PA; energy level; EMA-reported activity, location, mood, eating episodes, and social context; video use; Wi-Fi/Bluetooth disconnections.	EMA prompts 8 random times/day; goal-setting moments; progress-check moments; 4 PM check for no PA goal; goal-achievement points; health-coach dashboard review.	Goal setting, PA prompts, feedback, videos, coach messages	EMA/goal-based if-then rules	8 EMA prompts/day; 4 PM prompt if no PA goal; reinforcing message after goal achievement; coach message about once/week.	Self-report-based tailoring, goal/progress-based tailoring, and coach- or staff-supported tailoring	12 weeks
Hiremath et al [34], 2019	Social cognitive theory	PA	Android smartphone; PHIRE app; LG-Urbane smartwatch accelerometer; Bluetooth wheel-rotation monitor; EMA prompts; Firebase cloud storage; custom desktop visualization software; surveys on PA, pain, fatigue, and related information.	Real-time wheelchair-based PA classification, energy expenditure, distance traveled, moderate-intensity PA bouts, prior PA patterns, daily goal, minutes remaining to goal, and participant characteristics entered into the app, including weight, height, age, gender, and injury level.	PA classification and PA-level estimation occurred every minute. JITAI feedback was triggered when ≥3 continuous minutes of moderate-intensity or higher PA were detected; congratulatory messages continued every minute until activity stopped, and additional messages were sent when daily goals were reached or exceeded. EMA assessment prompts were delivered 6 times/day.	PA feedback, positive reinforcement, congratulatory messages, adaptive goal feedback	Sensor/ML^f^-detected PA if-then rules	JITAI prompts after ≥3 continuous minutes of moderate-intensity or higher PA; messages repeated every minute while activity continued; EMA prompts 6 times/day.	Sensor-based and tailoring and goal/progress-based tailoring	3 months
Pellegrini et al [35], 2015	Not reported	PA and sedentary behavior	NEAT! smartphone app on participants’ Android phones; Shimmer wireless accelerometer worn at the waist for intervention triggering; ActiGraph 7164 accelerometer for sedentary behavior and PA assessment.	Consecutive sedentary time, sit-to-stand transition, active vs sedentary state, participant prompt response, and participant preference for noise or vibration prompt.	A decision point occurred when 20 minutes of consecutive sedentary time was detected. The sedentary counter restarted when a sit-to-stand transition was detected or the participant responded to the phone prompt. If the participant selected Stand but did not stand, reminders were repeated every 2 minutes.	Sedentary break prompt, stand/light-activity reminder, response options	Sedentary-bout-triggered if-then rules	Prompt after 20 minutes of consecutive sedentary time; additional reminders every 2 minutes if the user selected “Stand” but did not stand.	Sensor-based and tailoring	1 month
Thomas et al [36], 2015	Not reported	Sedentary behavior	Android smartphone; B-MOBILE app; smartphone onboard accelerometer; validated algorithm classifying behavior as sedentary behavior or nonsedentary behavior in 1-minute epochs; SenseWear Mini Armband/objective multisensor monitor for assessment in the B-MOBILE study.	Continuous sedentary minutes, sedentary behavior vs nonsedentary behavior state, walking-break condition, walking-break completion, total daily reminders after 5 and 10 minutes of continued sedentary behavior minutes, total daily PA minutes, and number of walking prompts met.	Participants reached condition-specific sedentary thresholds: 30, 60, or 120 continuous sedentary minutes. The app also triggered reminders after 5 and 10 minutes of continued sedentary behavior after a walking prompt.	Walking-break prompts, reminder prompts, feedback display	Sedentary-threshold if-then rules	Three conditions were tested: a 3-min walking break after 30 min of sedentary behavior, a 6-min walking break after 60 min of sedentary behavior, or a 12-min walking break after 120 min of sedentary behavior; additional reminders were delivered after 5 and 10 min if sedentary behavior continued.	Sensor-based and tailoring	21 days

a JITAI: just-in-time adaptive intervention.

b PA: physical activity.

c EMA: ecological momentary assessment.

d MVPA: moderate to vigorous physical activity.

e CVD: cardiovascular disease.

f ML: machine learning.

Engagement and implementation-related measures were inconsistently reported across studies. Reported measures included study retention, wearable-device use, EMA completion, app use, goal setting, message exposure and responsiveness, intervention-delivery fidelity, usability, and satisfaction. Four studies [33,34,36,39] reported quantitative engagement and delivery-fidelity data. Hietbrink et al [19] and Daryabeygi-Khotbehsara et al [38] mainly reported qualitative acceptability or development-stage feedback, while Golbus et al [28], Bai et al [29], Elnaggar et al [31], and Park et al [32] provided limited objective usage or response data. Further details are presented in Multimedia Appendix 2.

### Effectiveness of JITAIs in Reducing Sedentary Behavior and Promoting PA Among Patients With Chronic Disease

Of the 14 included studies, 7 objectively measured PA. Three RCTs [28,29,32] reported preliminary evidence of a positive effect of JITAIs on promoting PA. In the study by Golbus et al [28], participants’ 60-minute step counts significantly increased within the first 30 days following the intervention. In the study by Park et al [32], the intervention group had a higher mean daily step count compared to the control group (*P*=.02). Similarly, Bai et al [29] found that participants in the Fitbit plus health coaching group increased their daily step count (*P*<.01) and moderate to vigorous physical activity (MVPA) (*P*<.05), with an improvement in daily MVPA compared to those in the Fitbit-only group (*P*<.05). Four nonrandomized studies reported PA outcomes, with three demonstrating positive effects [30,34,35]. Studies by Hiremath et al [34] and Pellegrini et al [35] reported increases in PA levels compared to baseline (both *P*<.05). The study by Lau et al [30] also showed that a higher proportion of light and moderate to vigorous PA was recorded among participants receiving JITAIs. However, the study conducted by Sengupta et al [33] found no significant change in PA levels compared to baseline.

Three studies [29,35,36] objectively measured sedentary behavior, including 1 RCT and 2 nonrandomized studies. In the RCT conducted by Bai et al [29], participants in the Fitbit plus health coaching group preliminarily demonstrated a reduction in sitting time (*P*<.01). Among the 2 nonrandomized studies, both reported a reduction in sedentary time or behavior compared with baseline (*P*<.05) [35,36]. Further details are provided in Multimedia Appendix 3.

### Barriers and Facilitators of JITAIs in Promoting PA and Reducing Sedentary Behavior Among Patients With Chronic Disease

#### Barriers Related to Engagement and Use of JITAIs

**Figure 2.**
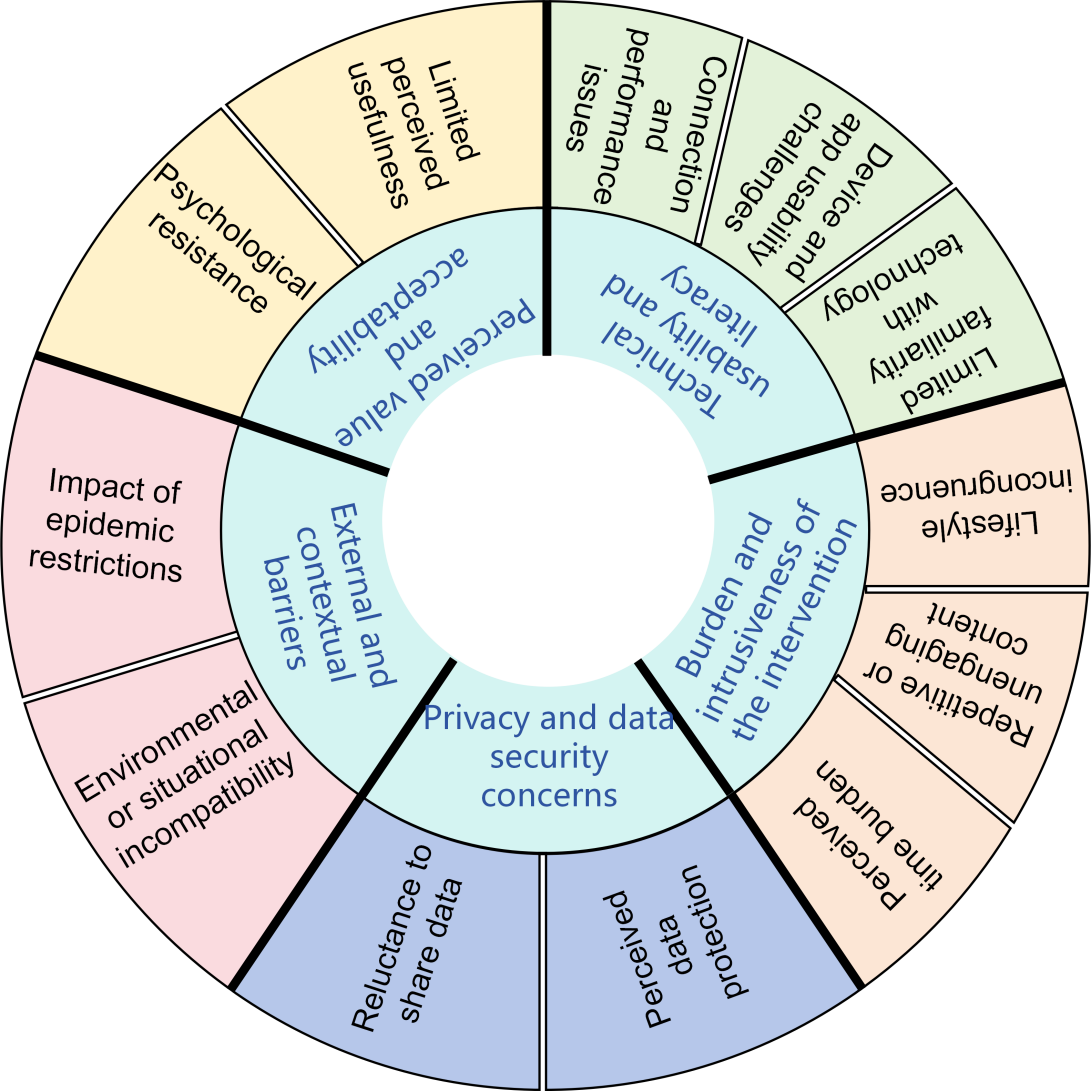
Barriers related to engagement and use of just-in-time adaptive interventions (JITAIs).

Several barriers were identified that may hinder the use of JITAIs in promoting PA and reducing sedentary behavior among patients with chronic diseases [19,20,31,34,37-39] (Figure 2). These barriers were categorized into five overarching themes: (1) technological usability and literacy, including limited familiarity with technology, device and app usability challenges such as small text or rigid settings, connection and performance issues such as devices unable to detect low-intensity or short-duration activities; (2) burden and intrusiveness of the intervention, including perceived time burden, repetitive or unengaging content, and lifestyle incongruence; (3) perceived value and acceptability, including limited perceived usefulness and psychological resistance; (4) external and contextual barriers, including the impact of epidemic restrictions and environmental or situational incompatibility; and (5) privacy and data security concerns, including reluctance to share data and perceived data protection.

#### Facilitators Related to Engagement and Use of JITAIs

**Figure 3.**
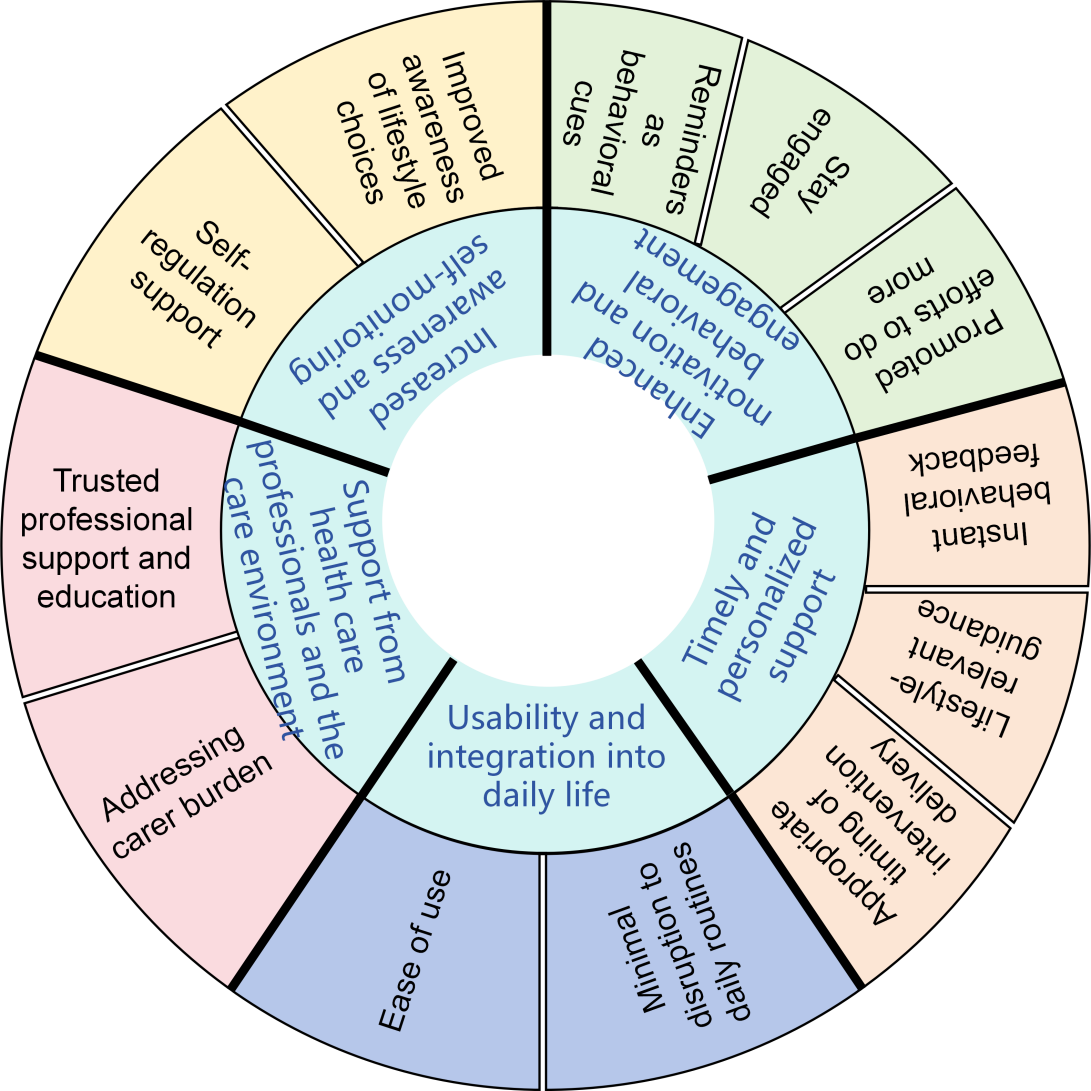
Facilitators related to engagement and use of just-in-time adaptive interventions (JITAIs).

The facilitators identified across the six included studies [19,31,34,37-39] were synthesized into five major themes: (1) enhanced motivation and behavioral engagement, including promoted efforts to “do more,” stay engaged and reminders as behavioral cues (eg, just-in-time reminders prompted timely action and supported formation of new routines and habits); (2) increased awareness and self-monitoring, including improved awareness of lifestyle choices and self-regulation support; (3) usability and integration into daily life, including ease of use and minimal disruption to daily routines; (4) timely and personalized support, including appropriate timing of intervention delivery (eg, participants felt supported “in the moment”), lifestyle-relevant guidance and instant behavioral feedback; and (5) support from health care professionals and the care environment, including trusted professional support and education, and addressing caregiver burden (Figure 3).

## Discussion

### Principal Findings

To the best of our knowledge, this is the first scoping review to systematically map and synthesize the existing literature on JITAIs specifically aimed at reducing sedentary behavior and promoting PA in individuals with chronic disease. A total of 14 studies were finally included in this review, and we systematically summarized the key characteristics of delivery methods and components used in JITAIs designed to reduce sedentary behavior and promote PA, and explored the existing evidence regarding the evaluation of these interventions in improving PA levels and reducing sedentary time. Additionally, this review also identified reported barriers and facilitators associated with the use of JITAIs among individuals living with chronic diseases. The findings contribute to a clearer understanding of how JITAIs are currently operationalized and provide a foundation for guiding future research and intervention development in chronic disease management.

Among the included studies, most of them provided participants with instructions on how to operate these technologies. As the integration of wearable activity trackers becomes more common, user training and support are essential to maximize engagement and minimize technological barriers [20,28]. The widespread use of built-in accelerometers and GPS sensors indicates that sensor-based context detection is a central feature of current JITAIs [30,35]. By identifying timely opportunities for intervention, these technologies may support more personalized and responsive behavioral prompts. Additionally, the intervention options and decision rules identified in this review suggest that most JITAIs used relatively simple designs. The frequent use of PA prompts, goal-setting or planning support, and feedback/encouragement indicates that most interventions focused on motivating participants to initiate or maintain PA, whereas fewer studies specifically targeted sedentary breaks. Safety prompts were reported only in Elnaggar et al [31] and Park et al [32], suggesting that symptom- or risk-related tailoring remains limited despite its relevance for patients with chronic disease. Decision rules were also mostly predefined and rule-based, which, although practical and transparent, indicates that many current JITAIs are adaptive mainly to predefined behavioral or contextual triggers rather than continuously learning from individual responses over time. Future research could therefore explore more dynamic and data-driven adaptation strategies that can continuously learn from users’ evolving behaviors and intervention responses.

In this review, most included studies reported preliminary evidence of a positive effect of JITAIs in promoting PA among patients with chronic disease. JITAIs provide personalized, real-time support based on individual context, such as location, time of day, or recent activity levels, which may enhance the relevance and timeliness of prompts and promote behavioral engagement [15,16]. The use of wearable sensors and mobile apps may also support continuous monitoring and feedback, potentially enhancing motivation and self-awareness [15,16]. In addition, many interventions incorporated behavior change techniques such as goal-setting, reminders, and feedback, which have been shown to support PA improvement [40]. However, the findings from one study [33] found no significant change in PA levels compared to baseline. One possible explanation for the lack of significant change in PA levels in the study could be the exclusive inclusion of female participants. Factors such as caregiving responsibilities, differing motivational drivers, and social or cultural expectations may influence women’s engagement with PA interventions [41]. Three studies [29,35,36] reported preliminary evidence of a positive effect of JITAIs in reducing sitting time and promoting breaks in sedentary behavior. The effects may be related to the real-time and context-sensitive design of JITAIs, which allows prompts or support to be delivered when individuals are likely to be sedentary, such as after prolonged inactivity or during sitting-related contexts, including watching television or working at a desk [29,35,36]. The timely cues may help interrupt habitual sedentary patterns and encourage individuals to take breaks, stand, or engage in light activity. However, the overall evidence base for JITAIs in promoting PA and reducing sedentary behavior remains limited by small sample sizes, few RCTs, and short intervention durations. In this review, only one study [28] had an intervention duration of 6 months, whereas the remaining studies involved relatively shorter intervention periods. The limited durations may not be sufficient to determine whether JITAIs can produce sustained behavioral changes or maintain long-term user engagement. Future research could therefore use RCT designs adequately, include longer intervention and follow-up periods, and recruit more diverse chronic disease populations across different clinical and community settings to further establish the effectiveness of JITAIs.

The identification of multiple barriers to JITAI use among patients with chronic diseases highlights important challenges that need to be addressed to optimize their utility. Most studies reported that technological usability and literacy emerged as a significant obstacle. Issues such as small text sizes, rigid app settings, and unreliable detection of low-intensity or brief activities can compromise user experience and reduce engagement, aligning with previous findings, which emphasize the digital divide, particularly among older or less technologically experienced populations commonly affected by chronic conditions [42,43]. Future interventions can adopt inclusive design principles that accommodate users with varying levels of digital literacy. This involves simplifying user interfaces, enabling customizable text sizes and display settings, and ensuring device compatibility with a wide range of PA levels, particularly low-intensity movements common among individuals with chronic diseases. Incorporating user-centered design through participatory development processes may enhance both usability and acceptability by aligning intervention features with patient needs and preferences [31,37]. Additionally, the burden and intrusiveness of the intervention represent another critical barrier. Patients reported perceptions of excessive time demands, repetitive or not engaging content, and misalignment with their daily routines, which may undermine adherence [20,34,38,39]. The results underscore the necessity for interventions that are not only effective but also seamlessly integrate into users’ lifestyles, minimizing disruption and maximizing relevance. Moreover, perceived value and acceptability also influence intervention uptake. Limited perceived usefulness and psychological resistance, potentially stemming from skepticism toward new technologies or concerns about personal capability, suggest that patient education and motivational strategies are crucial components for successful implementation [19]. Privacy and data security concerns were reported in several studies, reflecting widespread apprehension regarding data sharing and protection, similar to the findings from Muhunzi et al [44]. Future interventions should ensure transparent data policies and robust security measures while granting users control over their personal information to build trust, enhance sustained engagement, and support the further development of JITAIs.

### Limitations

This scoping review has several limitations that should be acknowledged. First, although a comprehensive search strategy was used, some relevant studies may have been missed due to publication bias or limitations in database indexing. In addition, only peer-reviewed articles published in English were included, which may have excluded relevant studies published in other languages or gray literature. Second, the heterogeneity of the included studies in terms of population characteristics, intervention designs, and duration limited our ability to quantitatively synthesize findings or make direct comparisons across studies. Third, many included studies had small sample sizes and short follow-up periods, which may limit the generalizability of the findings and make it difficult to determine whether the reported effects can be sustained over time. Additionally, most studies were conducted in high-income countries, which may limit the applicability of findings to low- and middle-income settings. Therefore, the conclusions should be interpreted with caution. Future systematic reviews with meta-analyses and risk-of-bias assessments are warranted to more rigorously evaluate the effectiveness of JITAIs in promoting PA and reducing sedentary behavior.

### Conclusions

This scoping review identified preliminary evidence suggesting that JITAIs may help reduce sedentary behavior and promote PA among patients with chronic diseases. However, the evidence supporting this conclusion remains limited by the small number of RCTs, small sample sizes, short intervention and follow-up periods, and heterogeneity across studies. Therefore, these findings should be interpreted as preliminary. Future research could use RCT designs with sufficient sample sizes, longer follow-up periods, and more diverse chronic disease populations to further establish the effectiveness and generalizability of JITAIs.

## Supplementary material

10.2196/81378 Multimedia Appendix 1 Search strategy.

10.2196/81378 Multimedia Appendix 2 Engagement and implementation-related measures of just-in-time adaptive interventions.

10.2196/81378 Multimedia Appendix 3 Effectiveness of just-in-time adaptive interventions among patients with chronic disease.

10.2196/81378 Checklist 1 PRISMA-ScR checklist.

## Abbreviations

EMA: ecological momentary assessment
JBI: Joanna Briggs Institute
JITAI: just-in-time adaptive intervention
mHealth: mobile health
MVPA: moderate to vigorous physical activity
PA: physical activity
PCC: population-concept-context framework
PRISMA-ScR: Preferred Reporting Items for Systematic Reviews and Meta-Analyses Extension for Scoping Reviews
RCT: randomized controlled trial
T2D: type 2 diabetes
