# Supplementary material for: Just-in-Time Adaptive Interventions to Reduce Sedentary Behavior and Promote Physical Activity in Patients With Chronic Disease: Scoping Review
Source: JMIR Mhealth Uhealth. 2026 Aug 7;14:e81378. doi: 10.2196/81378 (PMC13450892; doi:10.2196/81378)
Supplement: Supplementary file 2 [file mhealth-v14-e81378-s001.docx]

# Multimedia Appendix 1: Search strategy

| Database | Search strategy | Hits |
| --- | --- | --- |
| PubMed | #1 "chronic disease" [MeSH Terms] OR "essential hypertension" [MeSH Terms] OR "heart failure" [MeSH Terms] OR "coronary artery disease" [MeSH Terms] OR "stroke" [MeSH Terms] OR "paresis" [MeSH Terms] OR "arthritis" [MeSH Terms] OR "osteoarthritis" [MeSH Terms] OR "asthma" [MeSH Terms] OR "renal insufficiency, chronic" [MeSH Terms] OR "pulmonary disease, chronic obstructive" [MeSH Terms] OR "diabetes mellitus" [MeSH Terms] OR "insulin resistance" [MeSH Terms] OR "[hyperlipidemias](https://www.ncbi.nlm.nih.gov/mesh/68006949)" [MeSH Terms]  #2 "chronic disease" [Title/Abstract] OR "chronic illness" [Title/Abstract] OR "chronic condition" [Title/Abstract] OR "non communicable disease" [Title/Abstract] OR "noncommunicable disease" [Title/Abstract] OR "chronically ill" [Title/Abstract] OR "hypertension" [Title/Abstract] OR "hypertensive" OR "high blood pressure" [Title/Abstract] OR "heart failure" [Title/Abstract] OR "CHF" [Title/Abstract] OR "HF" [Title/Abstract] OR "cardiac failure" [Title/Abstract] OR "heart decompensation" [Title/Abstract] OR "coronary artery disease" [Title/Abstract] OR "coronary arteriosclerosis" [Title/Abstract] OR "coronary atherosclerosis" [Title/Abstract] OR "angina pectoris" [Title/Abstract] OR "CAD" [Title/Abstract] OR "heart disease" [Title/Abstract] OR "myocardial infarction" [Title/Abstract] OR "unstable angina*" [Title/Abstract] OR "angor pectoris" [Title/Abstract] OR "coronary thrombosis" [Title/Abstract] OR "acute coronary syndrome" [Title/Abstract] OR "myocardial ischemia" [Title/Abstract] OR "myocardial ischaemia" [Title/Abstract] OR "stroke" [Title/Abstract] OR "hemiplegia" [Title/Abstract] OR "hemiplegia" [Title/Abstract] OR "hemiplegias" [Title/Abstract] OR "paresis" [Title/Abstract] OR "cerebrovascular trauma" [Title/Abstract] OR "cerebrovascular accident" [Title/Abstract] OR "CVA" [Title/Abstract] OR "apoplexy" [Title/Abstract] OR "arthritis" [Title/Abstract] OR "rheuma*" [Title/Abstract] OR "osteoarthritis" [Title/Abstract] OR "arthritides" [Title/Abstract] OR "polyarthritis" [Title/Abstract] OR "polyarthritides" [Title/Abstract] OR "asthma" [Title/Abstract] OR "status asthmaticus" [Title/Abstract] OR "bronchial hyper reactivity" [Title/Abstract] OR "asthmatic" [Title/Abstract] OR "wheez" [Title/Abstract] OR "bronchial" [Title/Abstract] OR "obstructive lung disease"[Title/Abstract] OR "chronic renal insufficiency" [Title/Abstract] OR "chronic kidney failure" [Title/Abstract] OR "chronic renal failure" [Title/Abstract] OR "chronic renal disease" [Title/Abstract] OR "chronic kidney disease" [Title/Abstract] OR "chronic kidney disorder" [Title/Abstract] OR "CKD" [Title/Abstract] OR "ESRD" [Title/Abstract] OR "CRD" [Title/Abstract] OR "chronic kidney insufficiency" [Title/Abstract] OR "chronic obstructive pulmonary disease" [Title/Abstract] OR "chronic bronchitis" [Title/Abstract] OR "COPD" [Title/Abstract] OR "chronic obstructive airway disease" [Title/Abstract] OR "chronic airflow obstruction" [Title/Abstract] OR "chronic obstructive lung disease" [Title/Abstract] OR "emphysema" [Title/Abstract] OR "diabetes mellitus" [Title/Abstract] OR "insulin resistance" [Title/Abstract] OR "DMII" [Title/Abstract] OR "DM2" [Title/Abstract] OR "noninsulin dependent" [Title/Abstract] OR "impaired glucose tolerance" [Title/Abstract] OR "impaired glucose tolerant" [Title/Abstract] OR "[hyperlipidemias](https://www.ncbi.nlm.nih.gov/mesh/68006949)" [Title/Abstract] OR "overweight" [Title/Abstract] OR "obese" [Title/Abstract]  #3 #1 OR #2  #4 "motor activity" [MeSH Terms] OR "exercise" [MeSH Terms] OR "walking" [MeSH Terms] OR "sports" [MeSH Terms] OR "jogging" [MeSH Terms] OR "[swimming](https://www.ncbi.nlm.nih.gov/mesh/68013550)" [MeSH Terms] OR "health behaviour" [MeSH Terms] OR "cool-down exercise" [MeSH Terms] OR "gymnastics" [MeSH Terms] OR "muscle stretching exercises" [MeSH Terms] OR "circuit-based exercise" [MeSH Terms] OR "endurance training" [MeSH Terms] OR "high-intensity interval training" [MeSH Terms] OR "plyometric exercise" [MeSH Terms] OR "resistance training" [MeSH Terms] OR "warm-up exercise" [MeSH Terms] OR "weight lifting" [MeSH Terms] OR "screen time" [MeSH Terms]  #5 "physical activit*" [Title/Abstract] OR "physical fitness" [Title/Abstract] OR "sedentary behaviour" [Title/Abstract] OR "sitting" [Title/Abstract] OR "sedentariness" [Title/Abstract] OR "sedentar*" [Title/Abstract] OR "walk*" [Title/Abstract] OR "run" [Title/Abstract] OR "sport*" [Title/Abstract] OR "jog" [Title/Abstract] OR "swim*" [Title/Abstract] OR "cool-down exercise" [Title/Abstract] OR "cool down exercise" [Title/Abstract] OR "gymnastics" [Title/Abstract] OR "muscle stretching exercises" [Title/Abstract] OR "circuit-based exercise" [Title/Abstract] OR "endurance training" [Title/Abstract] OR "high-intensity interval training" [Title/Abstract] OR "high intensity interval train*" [Title/Abstract] OR "plyometric exercise" [Title/Abstract] OR "resistance training" [Title/Abstract] OR "resistance train*" [Title/Abstract] OR "warm-up exercise" [Title/Abstract] OR "weight lift*" [Title/Abstract] OR "circuit train*" [Title/Abstract] OR "circuit exercise*" [Title/Abstract] OR "weight train*" [Title/Abstract] OR "aerobic train*" [Title/Abstract] OR "aerobic exercise*" [Title/Abstract] OR "cardio train*" [Title/Abstract] OR "screen behavio*" [Title/Abstract] OR "screen Time" [Title/Abstract] OR "screen watching" [Title/Abstract] OR "TV watching" [Title/Abstract] OR "prolonged sitting" [Title/Abstract] OR "lying time" [Title/Abstract] OR "television viewing" [Title/Abstract] OR "active recreation" [Title/Abstract] OR "active leisure" [Title/Abstract] OR "active living" [Title/Abstract]  #6 #4 OR #5  #7 "Just-in-time adaptive intervention" [Title/Abstract] OR "Just-in-time" [Title/Abstract] OR "JITAI" [Title/Abstract] OR "ecological momentary intervention*" [Title/Abstract] OR "EMI" [Title/Abstract] OR "adaptive intervention" [Title/Abstract] OR "dynamic intervention" [Title/Abstract] OR "real time intervention*" [Title/Abstract] OR "context aware*" [Title/Abstract] OR "context triggered" [Title/Abstract] OR "context tailor*" [Title/Abstract] OR "dynamic tailor*" [Title/Abstract] OR "real time tailor*" [Title/Abstract] OR "sensor triggered" [Title/Abstract] OR "geofenc*" [Title/Abstract] OR "context sens*" [Title/Abstract] OR "real time context*" [Title/Abstract] OR "persuasive technolog*" [Title/Abstract] OR "sensing technolog*" [Title/Abstract]  #8 #3 AND #6 AND #7 | 165 |
| Web of science | #1 "chronic disease" [Title] OR "chronic illness" [Title] OR "chronic condition" [Title] OR "non communicable disease" [Title] OR "noncommunicable disease" [Title] OR "chronically ill" [Title] OR "hypertension" [Title] OR "hypertensive" OR "high blood pressure" [Title] OR "heart failure" [Title] OR "CHF" [Title] OR "HF" [Title] OR "cardiac failure" [Title] OR "heart decompensation" [Title] OR "coronary artery disease" [Title] OR "coronary arteriosclerosis" [Title] OR "coronary atherosclerosis" [Title] OR "angina pectoris" [Title] OR "CAD" [Title] OR "heart disease" [Title] OR "myocardial infarction" [Title] OR "unstable angina*" [Title] OR "angor pectoris" [Title] OR "coronary thrombosis" [Title] OR "acute coronary syndrome" [Title] OR "myocardial ischemia" [Title] OR "myocardial ischaemia" [Title] OR "stroke" [Title] OR "hemiplegia" [Title] OR "hemiplegia" [Title] OR "hemiplegias" [Title] OR "paresis" [Title] OR "cerebrovascular trauma" [Title] OR "cerebrovascular accident" [Title] OR "CVA" [Title] OR "apoplexy" [Title] OR "arthritis" [Title] OR "rheuma*" [Title] OR "osteoarthritis" [Title] OR "arthritides" [Title] OR "polyarthritis" [Title] OR "polyarthritides" [Title] OR "asthma" [Title] OR "status asthmaticus" [Title] OR "bronchial hyper reactivity" [Title] OR "asthmatic" [Title] OR "wheez" [Title] OR "bronchial" [Title] OR "obstructive lung disease"[Title] OR "chronic renal insufficiency" [Title] OR "chronic kidney failure" [Title] OR "chronic renal failure" [Title] OR "chronic renal disease" [Title] OR "chronic kidney disease" [Title] OR "chronic kidney disorder" [Title] OR "CKD" [Title] OR "ESRD" [Title] OR "CRD" [Title] OR "chronic kidney insufficiency" [Title] OR "chronic obstructive pulmonary disease" [Title] OR "chronic bronchitis" [Title] OR "COPD" [Title] OR "chronic obstructive airway disease" [Title] OR "chronic airflow obstruction" [Title] OR "chronic obstructive lung disease" [Title] OR "emphysema" [Title] OR "diabetes mellitus" [Title] OR "insulin resistance" [Title] OR "DMII" [Title] OR "DM2" [Title] OR "noninsulin dependent" [Title] OR "impaired glucose tolerance" [Titlet] OR "impaired glucose tolerant" [Title] OR "[hyperlipidemias](https://www.ncbi.nlm.nih.gov/mesh/68006949)" [Title] OR "overweight" [Title] OR "obese" [Title]  #2 "physical activit*" [Topic] OR "physical fitness" [Topic] OR "sedentary behaviour" [Topic] OR "sitting" [Topic] OR "sedentariness" [Topic] OR "sedentar*" [Topic] OR "walk*" [Topic] OR "run" [Topic] OR "sport*" [Topic] OR "jog" [Topic] OR "swim*" [Topic] OR "cool-down exercise" [Topic] OR "cool down exercise" [Topic] OR "gymnastics" [Topic] OR "muscle stretching exercises" [Topic] OR "circuit-based exercise" [Topic] OR "endurance training" [Topic] OR "high-intensity interval training" [Topic] OR "high intensity interval train*" [Topic] OR "plyometric exercise" [Topic] OR "resistance training" [Topic] OR "resistance train*" [Topic] OR "warm-up exercise" [Topic] OR "weight lift*" [Topic] OR "circuit train*" [Topic] OR "circuit exercise*" [Topic] OR "weight train*" [Topic] OR "aerobic train*" [Topic] OR "aerobic exercise*" [Topic] OR "cardio train*" [Topic] OR "screen behavio*" [Topic] OR "screen Time" [Topic] OR "screen watching" [Topic] OR "TV watching" [Topic] OR "prolonged sitting" [Topic] OR "lying time" [Topic] OR "television viewing" [Topic] OR "active recreation" [Topic] OR "active leisure" [Topic] OR "active living" [Topic]  #3 "Just-in-time adaptive intervention" [Topic] OR "Just-in-time" [Topic] OR "JITAI" [Topic] OR "ecological momentary intervention*" [Topic] OR "EMI" [Topic] OR "adaptive intervention" [Topic] OR "dynamic intervention" [Topic] OR "real time intervention*" [Topic] OR "context aware*" [Topic] OR "context triggered" [Topic] OR "context tailor*" [Topic] OR "dynamic tailor*" [Topic] OR "real time tailor*" [Topic] OR "sensor triggered" [Topic] OR "geofenc*" [Topic] OR "context sens*" [Topic] OR "real time context*" [Topic] OR "persuasive technolog*" [Topic] OR "sensing technolog*" [Topic]  #4 #1 AND #2 AND #3 | 1509 |
| Embase(via OVID) | #1 ("chronic disease" OR "chronic illness" OR "chronic condition" OR "non communicable disease" OR "noncommunicable disease" OR "chronically ill" OR hypertension OR hypertensive OR "high blood pressure" OR "heart failure" OR CHF OR HF OR "cardiac failure" OR "heart decompensation" OR "coronary artery disease" OR "coronary arteriosclerosis" OR "coronary atherosclerosis" OR "angina pectoris" OR CAD OR "heart disease" OR "myocardial infarction" OR "unstable angina*" OR "angor pectoris" OR "coronary thrombosis" OR "acute coronary syndrome" OR "myocardial ischemia" OR "myocardial ischaemia" OR stroke OR hemiplegia OR hemiplegia OR hemiplegias OR paresis OR "cerebrovascular trauma" OR "cerebrovascular accident" OR CVA OR apoplexy OR arthritis OR rheuma* OR osteoarthritis OR arthritides OR polyarthritis OR polyarthritides OR asthma OR "status asthmaticus" OR "bronchial hyper reactivit"y OR asthmatic OR wheez OR bronchial OR "obstructive lung disease" OR "chronic renal insufficiency" OR "chronic kidney failure" OR "chronic renal failure" OR "chronic renal disease" OR "chronic kidney disease" OR "chronic kidney disorder" OR CKD OR ESRD OR CRD OR "chronic kidney insufficiency" OR "chronic obstructive pulmonary disease" OR "chronic bronchitis" OR COPD OR "chronic obstructive airway disease" OR "chronic airflow obstruction" OR "chronic obstructive lung disease" OR emphysema OR "diabetes mellitus" OR "insulin resistance" OR DMII OR DM2 OR "noninsulin dependent" OR "impaired glucose tolerance" OR "impaired glucose tolerant" OR "[hyperlipidemias](https://www.ncbi.nlm.nih.gov/mesh/68006949)" OR "overweight" OR "obese").ab,kw,ti.  #2 ("physical activit*" OR "physical fitness" OR "sedentary behaviour" OR sitting OR sedentariness OR sedentar* OR walk* OR run OR sport* OR jog OR swim* OR "cool-down exercise" OR "cool down exercise" OR gymnastics OR "muscle stretching exercises" OR "circuit-based exercise" OR "endurance training" OR "high-intensity interval training" OR "high intensity interval train*" OR "plyometric exercise" OR "resistance training" OR "resistance train*" OR "warm-up exercise" OR "weight lift*" OR "circuit train*" OR "circuit exercise*" OR "weight train*" OR "aerobic train*" OR "aerobic exercise*" OR "cardio train*" OR "screen behavio*" OR "screen Time" OR "screen watching" OR TV watching OR "prolonged sitting" OR "lying time" OR "television viewing" OR "active recreation" OR "active leisure" OR "active living").ab,kw,ti.  #3 ("Just-in-time adaptive intervention" OR "Just-in-time" OR JITAI OR "ecological momentary intervention*" OR EMI OR "adaptive intervention" OR "dynamic intervention" OR "real time intervention*" OR "context aware*" OR "context triggered" OR "context tailor*" OR "dynamic tailor*" OR "real time tailor*" OR "sensor triggered" OR geofenc* OR "context sens*" OR "real time context*" OR "persuasive technolog*" OR "sensing technolog*").ab,kw,ti.  #4 #1 AND #2 AND #3 | 53 |
| CINAHL(via EBSCOhost Research Databases Interface） | #1 "chronic disease" [Title and Abstract] OR "chronic illness" [Title and Abstract] OR "chronic condition" [Title and Abstract] OR "non communicable disease" [Title and Abstract] OR "noncommunicable disease" [Title and Abstract] OR "chronically ill" [Title and Abstract] OR "hypertension" [Title and Abstract] OR "hypertensive" OR "high blood pressure" [Title and Abstract] OR "heart failure" [Title and Abstract] OR "CHF" [Title and Abstract] OR "HF" [Title and Abstract] OR "cardiac failure" [Title and Abstract] OR "heart decompensation" [Title and Abstract] OR "coronary artery disease" [Title and Abstract] OR "coronary arteriosclerosis" [Title and Abstract] OR "coronary atherosclerosis" [Title and Abstract] OR "angina pectoris" [Title and Abstract] OR "CAD" [Title and Abstract] OR "heart disease" [Title and Abstract] OR "myocardial infarction" [Title and Abstract] OR "unstable angina*" [Title and Abstract] OR "angor pectoris" [Title and Abstract] OR "coronary thrombosis" [Title and Abstract] OR "acute coronary syndrome" [Title and Abstract] OR "myocardial ischemia" [Title and Abstract] OR "myocardial ischaemia" [Title and Abstract] OR "stroke" [Title and Abstract] OR "hemiplegia" [Title and Abstract] OR "hemiplegia" [Title and Abstract] OR "hemiplegias" [Title and Abstract] OR "paresis" [Title and Abstract] OR "cerebrovascular trauma" [Title and Abstract] OR "cerebrovascular accident" [Title and Abstract] OR "CVA" [Title and Abstract] OR "apoplexy" [Title and Abstract] OR "arthritis" [Title and Abstract] OR "rheuma*" [Title and Abstract] OR "osteoarthritis" [Title and Abstract] OR "arthritides" [Title and Abstract] OR "polyarthritis" [Title and Abstract] OR "polyarthritides" [Title and Abstract] OR "asthma" [Title and Abstract] OR "status asthmaticus" [Title and Abstract] OR "bronchial hyper reactivity" [Title and Abstract] OR "asthmatic" [Title and Abstract] OR "wheez" [Title and Abstract] OR "bronchial" [Title and Abstract] OR "obstructive lung disease"[Title and Abstract] OR "chronic renal insufficiency" [Title and Abstract] OR "chronic kidney failure" [Title and Abstract] OR "chronic renal failure" [Title and Abstract] OR "chronic renal disease" [Title and Abstract] OR "chronic kidney disease" [Title and Abstract] OR "chronic kidney disorder" [Title and Abstract] OR "CKD" [Title and Abstract] OR "ESRD" [Title and Abstract] OR "CRD" [Title and Abstract] OR "chronic kidney insufficiency" [Title and Abstract] OR "chronic obstructive pulmonary disease" [Title and Abstract] OR "chronic bronchitis" [Title and Abstract] OR "COPD" [Title and Abstract] OR "chronic obstructive airway disease" [Title and Abstract] OR "chronic airflow obstruction" [Title and Abstract] OR "chronic obstructive lung disease" [Title and Abstract] OR "emphysema" [Title and Abstract] OR "diabetes mellitus" [Title and Abstract] OR "insulin resistance" [Title and Abstract] OR "DMII" [Title and Abstract] OR "DM2" [Title and Abstract] OR "noninsulin dependent" [Title and Abstract] OR "impaired glucose tolerance" [Title and Abstract] OR "impaired glucose tolerant" [Title and Abstract] OR "[hyperlipidemias](https://www.ncbi.nlm.nih.gov/mesh/68006949)" [Title and Abstract] OR "overweight" [Title and Abstract] OR "obese" [Title and Abstract]  #2 "physical activit*" [Title and Abstract] OR "physical fitness" [Title and Abstract] OR "sedentary behaviour" [Title and Abstract] OR "sitting" [Title and Abstract] OR "sedentariness" [Title and Abstract] OR "sedentar*" [Title and Abstract] OR "walk*" [Title and Abstract] OR "run" [Title and Abstract] OR "sport*" [Title and Abstract] OR "jog" [Title and Abstract] OR "swim*" [Title and Abstract] OR "cool-down exercise" [Title and Abstract] OR "cool down exercise" [Title and Abstract] OR "gymnastics" [Title and Abstract] OR "muscle stretching exercises" [Title and Abstract] OR "circuit-based exercise" [Title and Abstract] OR "endurance training" [Title and Abstract] OR "high-intensity interval training" [Title and Abstract] OR "high intensity interval train*" [Title and Abstract] OR "plyometric exercise" [Title and Abstract] OR "resistance training" [Title and Abstract] OR "resistance train*" [Title and Abstract] OR "warm-up exercise" [Title and Abstract] OR "weight lift*" [Title and Abstract] OR "circuit train*" [Title and Abstract] OR "circuit exercise*" [Title and Abstract] OR "weight train*" [Title and Abstract] OR "aerobic train*" [Title and Abstract] OR "aerobic exercise*" [Title and Abstract] OR "cardio train*" [Title and Abstract] OR "screen behavio*" [Title and Abstract] OR "screen Time" [Title and Abstract] OR "screen watching" [Title and Abstract] OR "TV watching" [Title and Abstract] OR "prolonged sitting" [Title and Abstract] OR "lying time" [Title and Abstract] OR "television viewing" [Title and Abstract] OR "active recreation" [Title and Abstract] OR "active leisure" [Title and Abstract] OR "active living" [Title and Abstract]  #3 "Just-in-time adaptive intervention" [Title and Abstract] OR "Just-in-time" [Title and Abstract] OR "JITAI" [Title and Abstract] OR "ecological momentary intervention*" [Title and Abstract] OR "EMI" [Title and Abstract] OR "adaptive intervention" [Title and Abstract] OR "dynamic intervention" [Title and Abstract] OR "real time intervention*" [Title and Abstract] OR "context aware*" [Title and Abstract] OR "context triggered" [Title and Abstract] OR "context tailor*" [Title and Abstract] OR "dynamic tailor*" [Title and Abstract] OR "real time tailor*" [Title and Abstract] OR "sensor triggered" [Title and Abstract] OR "geofenc*" [Title and Abstract] OR "context sens*" [Title and Abstract] OR "real time context*" [Title and Abstract] OR "persuasive technolog*" [Title and Abstract] OR "sensing technolog*" [Title and Abstract]  #4 #1 AND #2 AND #3 | 27 |
| Scopus | Search within: Article title, Abstract, Keywords  #1:  TITLE-ABS-KEY ("chronic disease" OR "chronic illness" OR "chronic condition" OR "non communicable disease" OR "noncommunicable disease" OR "chronically ill" OR hypertension OR hypertensive OR "high blood pressure" OR "heart failure" OR CHF OR HF OR "cardiac failure" OR "heart decompensation" OR "coronary artery disease" OR "coronary arteriosclerosis" OR "coronary atherosclerosis" OR "angina pectoris" OR CAD OR "heart disease" OR "myocardial infarction" OR "unstable angina*" OR "angor pectoris" OR "coronary thrombosis" OR "acute coronary syndrome" OR "myocardial ischemia" OR "myocardial ischaemia" OR stroke OR hemiplegia OR hemiplegia OR hemiplegias OR paresis OR "cerebrovascular trauma" OR "cerebrovascular accident" OR CVA OR apoplexy OR arthritis OR rheuma* OR osteoarthritis OR arthritides OR polyarthritis OR polyarthritides OR asthma OR "status asthmaticus" OR "bronchial hyper reactivit"y OR asthmatic OR wheez OR bronchial OR "obstructive lung disease" OR "chronic renal insufficiency" OR "chronic kidney failure" OR "chronic renal failure" OR "chronic renal disease" OR "chronic kidney disease" OR "chronic kidney disorder" OR CKD OR ESRD OR CRD OR "chronic kidney insufficiency" OR "chronic obstructive pulmonary disease" OR "chronic bronchitis" OR COPD OR "chronic obstructive airway disease" OR "chronic airflow obstruction" OR "chronic obstructive lung disease" OR emphysema OR "diabetes mellitus" OR "insulin resistance" OR DMII OR DM2 OR "noninsulin dependent" OR "impaired glucose tolerance" OR "impaired glucose tolerant")  #2 TITLE-ABS-KEY ("physical activit*" OR "physical fitness" OR "sedentary behaviour" OR sitting OR sedentariness OR sedentar* OR walk* OR run OR sport* OR jog OR swim* OR "cool-down exercise" OR "cool down exercise" OR gymnastics OR "muscle stretching exercises" OR "circuit-based exercise" OR "endurance training" OR "high-intensity interval training" OR "high intensity interval train*" OR "plyometric exercise" OR "resistance training" OR "resistance train*" OR "warm-up exercise" OR "weight lift*" OR "circuit train*" OR "circuit exercise*" OR "weight train*" OR "aerobic train*" OR "aerobic exercise*" OR "cardio train*" OR "screen behavio*" OR "screen Time" OR "screen watching" OR TV watching OR "prolonged sitting" OR "lying time" OR "television viewing" OR "active recreation" OR "active leisure" OR "active living")  #3 TITLE-ABS-KEY ("Just-in-time adaptive intervention" OR "Just-in-time" OR JITAI OR "ecological momentary intervention*" OR EMI OR "adaptive intervention" OR "dynamic intervention" OR "real time intervention*" OR "context aware*" OR "context triggered" OR "context tailor*" OR "dynamic tailor*" OR "real time tailor*" OR "sensor triggered" OR geofenc* OR "context sens*" OR "real time context*" OR "persuasive technolog*" OR "sensing technolog*")  #4 #1 AND #2 AND #3 | 59 |
