# Supplementary material for: Just-in-Time Adaptive Interventions to Reduce Sedentary Behavior and Promote Physical Activity in Patients With Chronic Disease: Scoping Review
Source: JMIR Mhealth Uhealth. 2026 Aug 7;14:e81378. doi: 10.2196/81378 (PMC13450892; doi:10.2196/81378)
Supplement: Supplementary file 3 [file mhealth-v14-e81378-s002.docx]

# Multimedia Appendix 2: Engagement and implementation-related measures of included studies

| Study | Engagement and implementation-related measures |
| --- | --- |
| Hietbrink et al [19], 2025 | Eight participants completed the 2-week intervention, while one participant withdrew after initially agreeing to participate. Interviews assessed EMA^a^ burden and response timing, message reading and responsiveness, perceived usefulness, and intention for continued use. Participants reported reading all messages. |
| Golbus et al [28], 2024 | Intervention exposure and delivery were reflected by the number of micro-randomizations: 108 participants contributed 70,552 randomizations over 6 months. Direct measures of message opening, response, or delivery success were not reported. |
| Novak et al [20], 2024 | Pilot implementation measures included the number and timing of messages delivered, Fitbit syncing and technical reliability, and participant feedback on usability, message content, frequency, and timing. Participants received a mean of 9.2 (SD 10.6) messages/week. |
| Bai et al [29], 2022 | 44 of 48 participants completed the study (91.7%). The majority of the participants were satisfied or very satisfied with the Fitbit (88.7%)  with no between-group differences.  A significantly higher percentage of participants from the Fitbit plus health coaching group perceived the Fitbit often or almost always motivating to be more active compared to the Fitbit (72.7% vs 45.5%, p < .05). The usage patterns, and intervention-delivery fidelity were not reported. |
| Daryabeygi-Khotbehsara et al [38], 2022 | Development-stage usability and implementation were assessed through six expert workshops and an end-user focus group (n=10), covering app navigation, functionality, aesthetics, content, connectivity, privacy, and willingness to use. Participants generally considered the app simple, useful, and easy to navigate. Formal usability testing and real-world adherence, usage, or delivery-fidelity measures were not reported. |
| Lau et al [30], 2022 | All participants completed the study. EMA and accelerometer compliance were 93.6% and 99.7%, respectively. Mean satisfaction was 4.8/5, confidence in using the system was 4.7/5, and preference over retrospective assessments was 4.7/5. |
| Mair et al [39], 2022 | 31 of 46 participants completed the study (67%), of whom 27/31 (87%) completed the intervention independently. Overall, 2247/2390 messages were successfully delivered (94%); Fitbit and weather data-call success rates were 99% and 100%, respectively. Completion rates were 50% for voice memos, 38% for mood assessments, and 50% for well-being assessments; 23/30 participants (77%) expressed satisfaction with the technology. |
| Elnaggar et al [31], 2021 | 7 of 26 intervention participants completed satisfaction questionnaires and interviews. Mean satisfaction scores were 4.86/5 for Fitbit, 4.50/5 for the Movn app, and 3.14/5 for push messages. Interviews assessed perceived usefulness, motivation, reminders, usability, and preferences for personalized feedback. |
| Park et al [32], 2021 | The retention rate was 85% (51/60). Adherence to the assigned intervention was verified through staff contact 3 days after enrolment and again after 1 month, and technical support was provided as needed. The overall acceptability by administering a satisfaction  questionnaire at the end of study with overall high satisfaction  scores for the Movn app and Fitbit device (4.5 and 4.86 out of  5, respectively). |
| Sengupta et al [33], 2020 | Among eight participants with engagement data, 132 goals were set, 830 EMA surveys were completed, eight educational videos were accessed 165 times, and 265 automated messages were delivered. Participants acknowledged a mean of 2.75 behavior-change messages/week. The mean System Usability Scale score was 83.6 (SD 16.3). |
| Hiremath et al [34], 2019 | 16 of 20 participants completed the study (80%). Adherence was monitored through smartwatch wear, smartphone use, device battery levels, and missing-data checks; participants were contacted after 3 consecutive days of missing data. Median EMA completion was 75%. Thirteen of 16 participants reported no app-use problems, 11/16 were willing to continue using the system, and all 16 would recommend it. |
| Pellegrini et al [35], 2015 | 8 of 9 participants completed the intervention (89%). Prompt-response behavior and technology acceptability were assessed. All completers reported increased awareness of their sitting time, and 7/8 (87.5%) expressed willingness to use the system in the future. |
| Thomas et al [36], 2015 | Participants carried the smartphone for a mean of 6.90 of the 7 days in each condition and for 14.94 h/day. Engagement measures included the number of walking prompts and completed walking breaks, the percentage of prompts resulting in the prescribed walking break, response latency, and time spent in prompted walking breaks. Prompt adherence decreased modestly over time. |

^a^EMA: ecological momentary assessment.
