# Supplementary material for: Just-in-Time Adaptive Interventions to Reduce Sedentary Behavior and Promote Physical Activity in Patients With Chronic Disease: Scoping Review
Source: JMIR Mhealth Uhealth. 2026 Aug 7;14:e81378. doi: 10.2196/81378 (PMC13450892; doi:10.2196/81378)
Supplement: Supplementary file 4 [file mhealth-v14-e81378-s003.docx]

# Multimedia Appendix 3: Effectiveness of JITAIs in reducing [sedentary behavior and promote P](https://www.webofscience.com/wos/woscc/full-record/WOS:000695742000003)A among patients with chronic disease

| Author | Year of publication | Findings |
| --- | --- | --- |
| Golbus et al [28] | 2024 | Amongst 40 Fitbit users, mean step count in the 60-minutes following a decision point was 625.3 (SD 863.3) steps. During the initiation period of the study, delivering a message increased 60-minute post-randomization step count by 17% (95% CI +7% to 28%; p＜0.001) |
| Lau et al [30] | 2022 | The moderate to vigorous physical activity time captured by the accelerometer was positively correlated with the moderate to vigorous physical activity time assessed by the International Physical Activity Questionnaire-Long Form (P=.001) |
| Bai et al [29] | 2022 | The FB+ group significantly increased PA^a^ [+1854 (2518) steps/day, p < .01] and MVPA [+26 (34) mins/day, p < .05], and decreased their ST [−63 (73) mins/day, p < .01]. The FB group significantly increased MVPA^b^ [+11 (16) mins/day, p < .05], and decreased their ST [−87 (117) mins/ day, p < .01]. |
| Park et al [32] | 2021 | The results from 51 patients who completed follow-up showed the intervention group had a statistically significant higher mean daily step count compared with the control (8860 vs 6633; P=.02) |
| Sengupta et al [33] | 2020 | The participants demonstrated statistically significant improvements in waist circumference (P=.048), weight (P=.02), and BMI^c^ (P=.01). The International Physical Activity Questionnaire showed no statistically significant improvements at baseline vs 12-week follow-up. |
| Hiremath et al [34] | 2019 | Eleven participants (69.0%) had higher light- and/or moderate-intensity PA during PA Feedback with JITAI^d^ compared to the baseline. |
| Pellegrini et al [35] | 2015 | Sedentary time decreased by 8.1±4.5 %, and light physical activity increased by 7.9±5.5 % over the 1-month period. |
| Thomas et al [36] | 2015 | The 3- and 6-min conditions resulted in the greatest number of prompts, walking breaks, the best adherence to prompts, the greatest amount of daily time spent in walking breaks, and fastest adherence to prompts (P< .01). |

^a^PA: physical activity; ^b^MVPA: moderate-to-vigorous physical activity; ^c^BMI: body mass index; ^d^JITAI: just-in-time adaptive intervention.
